# Surgical Intervention and Treatment of a Mammary Adenocarcinoma in the Sugar Glider (*Petaurus breviceps*): A Double Case Report

**DOI:** 10.1155/crve/6684002

**Published:** 2026-07-18

**Authors:** Taryn Peña, Zoë Tormollen

**Affiliations:** ^1^ North Austin Animal Hospital, Austin, Texas, USA

**Keywords:** case report, mammary adenocarcinoma, neoplasia, sugar glider, surgery

## Abstract

A 6‐year‐old, intact female sugar glider (*Petaurus breviceps*) presented for evaluation after experiencing progressive weight loss and lethargy. Sedated examination revealed a firm, 7‐mm wide marsupium mass, and surgery was pursued after cytology results suggested epithelial malignancy. The patient′s entire marsupium was surgically excised, and biopsy of the associated tumor revealed a primary mammary adenocarcinoma without vascular infiltration. Preoperative and postoperative antibiotics and meloxicam were used to decrease inflammation and infection following the procedure. At the 6‐ and 12‐month recheck examinations, the initial patient was well‐muscled and energetic with no signs of neoplastic recurrence. Case 1 had confirmed disease‐free survival at 12 months. A second 6‐year‐old, intact female sugar glider presented for surgical consultation following the fine‐needle aspiration of a bilobular marsupium mass. Analogous surgical protocols were implemented with the second patient. Subsequent biopsy revealed neoplastic epithelial cells consistent with a mammary adenocarcinoma. No local recurrence or disease progression has been reported for the second patient following the operation. Case 2 had owner‐reported well‐being at 11 months without clinical re‐examination. To the authors′ knowledge, these cases represent the first reported survival beyond 11 months following surgical treatment of mammary adenocarcinoma in *P. breviceps*, though long‐term recurrence risk remains unknown.

## 1. Introduction

Sugar gliders are nocturnal, arboreal marsupials native to Australia [1]. Though their popularity as a companion animal has grown in recent decades, neoplastic research remains limited in this species [2]. Only two mammary carcinoma case studies in *Petaurus breviceps* were identified prior to this report, and both patients were euthanized due to disease progression or complications [3, 4]. In comparison, the patients detailed in this article are alive with no reported local neoplastic recurrence > 11 months after their respective treatments.

## 2. Case Presentation One

A 6‐year‐old, 89‐g, intact female sugar glider (*P. breviceps*) presented for examination after owners noticed acute alopecia over the left shoulder. The patient was housed indoors in a 1.8‐m tall, wire enclosure with two other sugar gliders. The patient′s diet consisted of eggs, honey, baby food, wheat germ, yogurt, and vitamins. A 5‐mm full skin laceration along the left shoulder was discovered on examination. Diffuse muscle atrophy was noted, and a body condition score of 3.5/9.0 was recorded. The laceration was suspected to be traumatic, and the patient was started on trimethoprim and sulfamethoxazole (30 mg/kg, orally, every 12 h for 7 days, TMS; Novitium Pharmacy, LLC., East Windsor, New Jersey, United States) and meloxicam (0.2 mg/kg, orally, every 24 h for 7 days; Covetrus, Dublin, Ohio, United States).

On Day 34 after the initial presentation, the patient returned for evaluation. The laceration had resolved, but the muscle atrophy had progressed. The animal had become dull and inappetent. A body condition score of 3.0/9.0 was reported. On Day 49, the patient returned for a sedated examination after failing to improve. Midazolam (0.5 mg/kg; Hospira, Inc., Lake Forest, Illinois, United States) and buprenorphine (0.04 mg/kg; Wedgewood Pharmacy, LLC., San Jose, California, United States) were injected intramuscularly for premedication. Induction and maintenance were achieved through the administration of 4% sevoflurane gas (Covetrus, Dublin, Ohio, United States) mixed with 100% oxygen (2 L/min). Sedation allowed for an extensive physical examination, where a firm, 7‐mm mass was discovered within the marsupium. The mass was sampled via fine‐needle aspiration, and the acquired cellular material was submitted for cytology. The patient recovered uneventfully from anesthesia.

The cytology revealed numerous clusters of epithelial cells interspersed with neutrophils and frequent foamy mononuclear cells. The epithelial cells had a high nuclear‐to‐cytoplasmic ratio and often indistinct cell borders. Many of the cells lacked distinct nucleoli. Mammary neoplasia such as an adenoma or adenocarcinoma was suspected by the pathologist. Given this information, an abdominal ultrasound was recommended to assess the patient′s surgical suitability. A sedated ultrasound was performed at another facility 7 days later. Although the presence of hyperechoic foci in the liver did increase concern for metastasis, aspirates of the foci were declined by the owners. At this point, the owners elected surgical intervention.

On Day 63 following the initial examination, the patient presented for surgery. Midazolam (0.5 mg/kg) and buprenorphine (0.04 mg/kg) were injected intramuscularly for premedication. Induction and maintenance were achieved through the administration of 3% sevoflurane gas mixed with 100% oxygen (2 L/min). Heat support was provided throughout, and vitals were monitored continuously. The patient was placed into ventral recumbency. A firm, circular mass was noted within the pouch. The region was clipped with a 40 blade (Covetrus, Dublin, Ohio, United States), and the area was surgically prepped with 2% chlorhexidine gluconate scrub (Covetrus, Dublin, Ohio, United States) and rinsed with dilute alcohol. An elliptical incision was made around the pouch. Using blunt dissection, the marsupium and associated mass were elevated. The vessels were ligated with 3‐0 monocryl (Ethicon; LLC., Guaynabo, Puerto Rico, United States) and transected. The marsupium and mass were removed from the body. The subcutaneous tissue was closed with 3‐0 monocryl in a simple, continuous pattern. The skin was closed with the same suture in an intradermal pattern. Meloxicam (0.2 mg/kg, subcutaneously, Metacam; Boehringer Ingelheim, Inc., Duluth, Georgia, United States) was injected perioperatively, and the patient recovered uneventfully. The mass was preserved in 10% buffered formalin (Formalin; VWR International, LLC., Radnor, Pennsylvania, United States) following standard sample protocol, and submitted for biopsy. The patient was discharged the same day and sent home with a restraint jacket to mitigate self‐mutilation behaviors. Meloxicam (0.2 mg/kg, orally, every 24 h for 7 days) and gabapentin (5 mg/kg, orally, every 12–24 h for 7 days; Amneal Pharmaceuticals, LLC., Bridgewater, New Jersey, United States) were started the next day.

**Figure 1:**
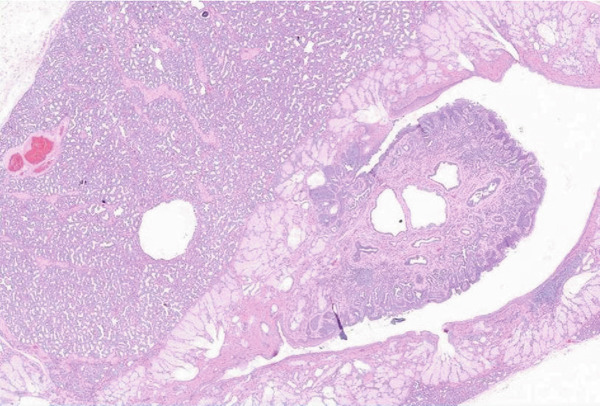
Histopathological image of a mammary adenocarcinoma in a female, intact sugar glider. H&E. Reproduced with permission from IDEXX laboratories [5].

**Figure 2:**
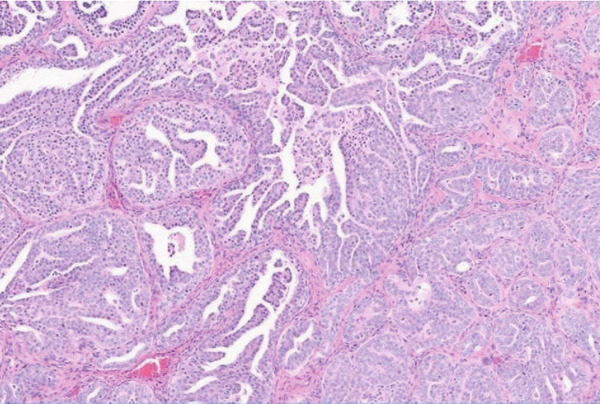
Histopathological image of the mammary adenocarcinoma architecture. H&E. Reproduced with permission from IDEXX laboratories [5].

The submitted tissue consisted of haired skin with subjacent bilateral pouches. On one side, the pouch was surrounded by a rim of glandular tissue with clusters of sebaceous‐type glands closest to the proximal lumen. Further surrounding the layer of sebaceous‐type glands was a thick rim of mammary gland tissue. Segmentally, both the sebaceous‐glandular tissue and mammary tissue were effaced by neoplastic proliferation. The neoplastic mass was well demarcated and consisted of highly irregular tubular structures with frequent intraluminal papillary projections (Figure 1). Individual neoplastic cells were polygonal with indistinct borders and typically contained a small‐to‐moderate amount of eosinophilic cytoplasm (Figure 2). Nuclei were ovular with stippled chromatin and occasionally had 1+ small nucleoli. Anisocytosis and anisokaryosis were mild‐to‐moderate, and there were 40 mitotic figures in 2.37 mm^2^. Small foci with very early infiltrative behavior were present.

It was concluded that the tissue originated from a unilateral mammary adenocarcinoma with contralateral mammary gland hyperplasia. Neoplastic cells were < 1 mm from the peripheral and deep edges of the tissue submitted. No vascular invasion was present. Narrow surgical margins were achieved.

On Day 7 following the procedure, the patient returned for evaluation. Owners reported self‐mutilation behavior and anorexia at home. An 8‐mm portion of the caudal incision was open, with purulent discharge and mild erythema. The patient was restarted on TMS (30 mg/kg, orally, every 12 h for 14 days). At the 2‐week follow‐up, the patient′s appetite had begun to improve. Examination of the incision revealed healthy tissue beginning to contract inwards, and no progression of the skin infection was evident. The patient had gained 1.5 g, which marked the first recorded weight increase since symptoms arose.

At the penultimate follow‐up (177 days following excisional surgery), the patient was bright, moderately well‐muscled, and had no visible signs of neoplastic recurrence. At the final examination (376 days following excisional surgery), the animal was energetic, well‐muscled, and showed no signs of disease progression. The patient was given a body condition score of 5.0/9.0.

## 3. Case Presentation Two

A second 6‐year‐old, intact female sugar glider presented for consultation after two marsupium‐associated tumors were detected at a previous clinic. The patient was housed in a 1.5‐m tall, wire‐framed enclosure with one other intact female glider. The animals′ diet consisted of High Protein Wombaroo powder (Instant HPW, Exotic Nutrition, Newport, Virginia, United States), honey, scrambled eggs, and bee pollen. On examination, the patient was euhydrated, appropriately muscled, and all vitals were within normal limits. Two firm masses were palpable in the marsupium: The smaller tumor was approximately 3 mm in diameter, whereas the larger tumor was approximately 5 mm in diameter. Cytology results from the previous clinic indicated that the tumors were suspected to be neoplastic and associated with the epithelium. Surgical excision was discussed at the examination, though medical management was offered as a more conservative approach. After several days of consideration, the owner elected surgical intervention. No preoperative imaging was performed to evaluate for metastatic disease, which was a limitation of this case.

On Day 13 following the consultation, the patient returned for surgery. Midazolam (0.5 mg/kg) and buprenorphine (0.04 mg/kg) were mixed together and injected intramuscularly for premedication. Induction and maintenance were achieved through the administration of 3.5% sevoflurane gas mixed with 100% oxygen (1 L/min). Heat support was provided throughout the procedure, and vitals were monitored continuously. The patient was placed into ventral recumbency. A firm mass with two lobes was noted within the pouch. The region was clipped with a 40 blade, and the area was surgically prepped with chlorhexidine scrub and rinsed with alcohol. An elliptical incision was made around the marsupium. Blunt dissection was performed with mosquito hemostats, and the marsupium and associated mass were elevated. The vessels were ligated with 3‐0 monocryl and transected. The marsupium and associated mass were removed from the body. The subcutaneous tissue was closed with the same suture in a simple, continuous pattern. The skin was closed in an intradermal pattern. Meloxicam (0.2 mg/kg) was injected subcutaneously and perioperatively for pain control. The tumor was preserved in 10% buffered formalin following standard sample protocol before being submitted for biopsy. The patient recovered uneventfully, and a restraint jacket was provided to mitigate self‐mutilation behaviors at home. Oral meloxicam (0.2 mg/kg, orally, every 24 h for 7 days) and gabapentin (5 mg/kg, orally, every 12 h for 14 days) were started the next morning. The surgical procedure and postoperative medication protocol were nearly identical to the previous mammary tumor case.

The submitted tissue consisted of cutaneous, haired skin along with the excised pouch and associated tumor. Biopsy of the tissue revealed raised proliferations of epithelial cells arranged in acini and tubules: the epithelial cells had indistinct cell borders, small amounts of eosinophilic cytoplasm, and round nuclei with stippled chromatin. Moderate cellular pleomorphism was noted, indicating neoplastic progression within the demarcated region. Mild peripheral infiltration was apparent and the surface of the tumor was regionally ulcerated. There were seven mitotic figures in 2.37 mm^2^ (approximately 10 high‐powered fields) and seven histologic tumor‐free margins with clear but narrow demarcation at the deep margin (within 1 mm). The nearest peripheral margin was 7 mm in width. No vascular invasion was observed. Definitive mammary origin could not be confirmed without immunohistochemical markers, though the location and histologic appearance strongly suggested a mammary adenocarcinoma.

On Day 5 following the procedure, a postoperative recheck was performed. On examination, the patient was moderately well‐muscled, the incision was healing appropriately, and vitals were within normal parameters.

On Day 17 following the procedure, the final examination was performed. Reportedly, the patient′s appetite and energy were normal at home. The patient was euhydrated, bright, responsive, and vitals were within normal limits. No follow‐up visits were scheduled or advised. On Day 337 following the procedure, a phone consultation revealed the patient was doing well with no reported concerns.

## 4. Discussion

Mammary carcinomas have been documented in a variety of animals including humans, felines, canines, rodents, hedgehogs, and marsupials [2, 3, 6–12]. The survival periods of afflicted animals are largely variable, with factors such as species, tumor size, lymphatic involvement, and clinical presentation playing a role in the variance [6, 7, 13]. Differences in neoplastic behavior and lack of standardization among studies have made reliable prognosis difficult in afflicted companion animals [7, 9, 13].

After an extensive literary search by the authors, only two mammary carcinoma studies were identified in *P. breviceps* prior to this article, and both reported unfavorable outcomes [3, 4]. Additionally, only one of the two cases described a mammary adenocarcinoma [3]. On a broader scope, neoplastic literature in this species is quite limited. A retrospective review, which included cases in *P. breviceps* that described a specific neoplasm, basic patient information, and a pursued treatment, found that only eight published case reports met these aforementioned criteria [2–4, 14–19].

Based on the limited literature, mammary carcinomas appear to be relatively aggressive in this species. In one 2015 study, a 9‐year‐old sugar glider was diagnosed with a mammary adenoma following the aspiration of a small marsupium‐associated mass [3]. Surgical treatment was not pursued, and the patient was euthanized approximately 7 months later due to unilateral hind‐limb paralysis [3]. Subsequent necropsy revealed a primary mammary adenocarcinoma with metastasis to the axillary lymph nodes, lungs, and spinal cord [3]. While one 2014 study described the surgical resection of an anaplastic mammary carcinoma with no perioperative complications, the sugar glider was euthanized 10 days later due to quality of life concerns [4]. Prior to the subjects described in this paper, there have been no known reports of prolonged survival (> 10 days) in sugar gliders following treatment for a mammary carcinoma [3, 4]. At the time of submission, both marsupials described in this study have been alive for > 11 months following their respective surgeries. Case 1 had confirmed disease‐free survival at 12 months. Case 2 had owner‐reported well‐being at 11 months without clinical re‐examination.

Additionally, a 2022 review, which surveyed eight comprehensive reports on sugar glider neoplasia, found that just two animals achieved remission following cancer treatment [2–4, 14–19]. The two cases included a dermal hemangiosarcoma in 2014 and a paracloacal gland carcinoma in 2018 [2, 16, 18]. In both patients, antibiotic and anti‐inflammatory courses were initiated before moving to surgical excision of the neoplastic tissue [16, 18]. Following surgery, antibiotics were utilized in conjunction with meloxicam to treat lingering infection and inflammation [16, 18]. Similar methodologies were utilized for the patients described in this report.

In the cases described in this study, the entire marsupium was removed in place of a local lumpectomy. Marsupium excision allowed for distinct surgical borders and narrow margins around the tumors. Whether complete marsupium excision offers advantages over local lumpectomy in sugar gliders is unknown and warrants further investigation.

Although cancer research in *P. breviceps* remains in its infancy, this study, and analogous cases, provide insight on areas deserving of further investigation. First, there is a need for more comprehensive reporting on neoplasia in small exotic animals. Second, to the authors′ knowledge, only retrospective studies exist in this species. Standardized trials would be invaluable in assessing adenocarcinoma characteristics and therapeutic efficacy. Cross‐species research is a well‐established practice in mammary carcinoma queries, with mice and canines often being used as models for human breast cancer [12, 13, 20]. Thus, a better understanding of mammary adenocarcinoma behavior has the potential to benefit patients of all backgrounds.

## Author Contributions

T.P.: conceptualization, methodology, validation, supervision, and project administration. Z.T.: writing—original draft, writing—review & editing, and visualization.

## Funding

No funding was received for this manuscript.

## Consent

Written consent was obtained from the respective owners for sedation, diagnostic testing, surgery, and the publication of this report.

## Conflicts of Interest

The authors declare no conflicts of interest.

## Supporting information


**Supporting Information** Additional supporting information can be found online in the Supporting Information section. CARE reporting guidelines were followed during the preparation of this report. A completed 2013 CARE checklist was included as supporting information upon submission.

## Data Availability

The data that supports the findings of this study are available from the corresponding author upon reasonable request.
