# Supplementary material for: Surgical Intervention and Treatment of a Mammary Adenocarcinoma in the Sugar Glider (Petaurus breviceps): A Double Case Report
Source: Case Rep Vet Med. 2026 Jul 18;2026:6684002. doi: 10.1155/crve/6684002 (PMC13379947; doi:10.1155/crve/6684002)
Supplement: Supplementary file 1 — Supporting Information Additional supporting information can be found online in the Supporting Information section. CARE reporting guidelines were followed during the preparation of this report. A completed 2013 CARE checklist was included as supporting information upon submission. [file CRVE-2026-6684002-s001.pdf]

CARE case report guidelines

CARE Checklist of information to include when writing a case report

(cc) BY-NC-ND

Topic	Item	Checklist item description	Reported on Line
**Title**	**1**	The diagnosis or intervention of primary focus followed by the words “case report”	1
**Key Words**	**2**	2 to 5 key words that identify diagnoses or interventions in this case report, including "case report"	63
**Abstract (no references)**	**3a**	Introduction: What is unique about this case and what does it add to the scientific literature?	59-61
	**3b**	Main symptoms and/or important clinical findings	48, 49, 55, 56
	**3c**	The main diagnoses, therapeutic interventions, and outcomes	50-54, 56-59
	**3d**	Conclusion—What is the main “take-away” lesson(s) from this case?	59-61
**Introduction**	**4**	One or two paragraphs summarizing why this case is unique (**may include references**)	67-72
**Patient Information**	**5a**	De-identified patient specific information.	76-79, 163-166
	**5b**	Primary concerns and symptoms of the patient.	86-89, 167, 168
	**5c**	Medical, family, and psycho-social history including relevant genetic information	N/A
	**5d**	Relevant past interventions with outcomes	79-82
**Clinical Findings**	**6**	Describe significant physical examination (PE) and important clinical findings.	86-100, 167-169
**Timeline**	**7**	Historical and current information from this episode of care organized as a timeline	76-209
**Diagnostic Assessment**	**8a**	Diagnostic testing (such as PE, laboratory testing, imaging, surveys).	93-104, 125-147, 194-200
	**8b**	Diagnostic challenges (such as access to testing, financial, or cultural)	103, 172-174, 201-203
	**8c**	Diagnosis (including other diagnoses considered)	99. 100. 145. 146. 170. 202. 203
	**8d**	Prognosis (such as staging in oncology) where applicable	102, 103, 157, 173, 210
**Therapeutic Intervention**	**9a**	Types of therapeutic intervention (such as pharmacologic, surgical, preventive, self-care)	81-83. 106-123. 151. 176-192
	**9b**	Administration of therapeutic intervention (such as dosage, strength, duration)	82. 83. 89. 106. 107. 117. 122. 151. 176. 186. 190. 191
	**9c**	Changes in therapeutic intervention (with rationale)	150, 151
**Follow-up and Outcomes**	**10a**	Clinician and patient-assessed outcomes (if available)	156-159, 209-212
	**10b**	Important follow-up diagnostic and other test results	N/A
	**10c**	Intervention adherence and tolerability (How was this assessed?)	149-154, 205-206
	**10d**	Adverse and unanticipated events	149-151
**Discussion**	**11a**	A scientific discussion of the strengths AND limitations associated with this case report	236-240, 250-258
	**11b**	Discussion of the relevant medical literature **with references**.	222-236, 242-248
	**11c**	The scientific rationale for any conclusions (including assessment of possible causes)	243-248
	**11d**	The primary “take-away” lessons of this case report (without references) in a one paragraph conclusion	255-262
**Patient Perspective**	**12**	The patient should share their perspective in one to two paragraphs on the treatment(s) they received	N/A
**Informed Consent**	**13**	Did the patient give informed consent? Please provide if requested (Owner Consent)	**Yes** ☒ **No** ☐
